# A circadian rhythm-related gene signature for prognosis, invasion and immune microenvironment of breast cancer

**DOI:** 10.3389/fgene.2022.1104338

**Published:** 2023-01-05

**Authors:** Mei-Huan Wang, Xiao Liu, Qian Wang, Hua-Wei Zhang

**Affiliations:** ^1^ Shandong Provincial Hospital Affiliated to Shandong First Medical University, Jinan, China; ^2^ Department of Ultrasound, Shandong Provincial Hospital, Shandong University, Jinan, Shandong, China

**Keywords:** circadian rhythm, breast cancer, prognosis, immunity, TCGA

## Abstract

**Background:** Circadian dysregulation is linked to the onset and progression of cancer, but current knowledge of the role of circadian rhythm-related genes (CRRGs) in breast cancer (BC) is limited and incomplete. The purpose of this study was to investigate the potential role and immune-related prognostic significance of CRRGs in BC.

**Methods:** The Cancer Genome Atlas breast cancer (TCGA-BRCA) genetic data were combined with 1369 CRRGs to create a model of BC prognosis-related CRRGs. To validate the model’s predictive power in TCGA and other external datasets, the Kaplan-Meier survival curve and receptor operation characteristic curve were plotted. The relationship between CRRGs model and gene enrichment pathways, immune cell infiltration, and differences in patient response to immune checkpoint inhibitors (ICIs) therapy was then discussed.

**Results:** A CRRG-based eighteen-gene model was developed that accurately predicted the survival time of BC patients. Based on this model, BC patients can be classified as high or low risk. The high-risk group has negative immune cell infiltration (such as macrophages M0 and M2) and a poor therapeutic response to ICIs due to lower immune checkpoint gene expression. Furthermore, TCF7 and IFNG were found to be strongly associated with immune checkpoints in CRRGs model.

**Conclusion:** The 18 CRRGs may be useful in assessing the prognosis of BC patients, studying immune infiltration, and developing more effective immunotherapy strategies.

## 1 Introduction

According to GLOBOCAN, the number of new cases of breast cancer has surpassed lung cancer as the world’s leading cancer, reaching 2.3 million in 2020 (Sung et al., 2021). Despite significant advancements in the treatment of BC, such as immunotherapy (Emens, 2018) and immune checkpoint inhibitors (ICIs) (Keenan and Tolaney, 2020). Due to the heterogeneity of BC, recurrence and metastasis remain the most typical reasons for treatment failure (Weigelt et al., 2005), and the 5-year survival rate of metastatic BC is just 26% (Peart, 2017). Therefore, identifying key molecular markers associated with the malignant transformation of BC cells and tumor progression is critical for effective BC diagnosis and prognosis prediction in BC patients.

Circadian rhythm is a 24-h cycle in which the hypothalamic suprachiasmatic nucleus (SCN) acts as a central clock to regulate the vital activities of the body (Koronowski and Sassone-Corsi, 2021). The core transcription-translation feedback loop (TTFL), which includes CLOCK, BMAL1, cryptochromes (CRY1, CRY2), and cyclins (PER1-3), generates circadian rhythm at the molecular level (Koronowski and Sassone-Corsi, 2021). CLOCK and BMAL1 heterodimers activate the transcription of PER and CRY in the morning by binding to the E-box response element within their promoters (Takahashi, 2017). Later in the day, PER and CRY proteins accumulate in the cytoplasm and transfer to the nucleus, where they bind and negatively regulate circadian mechanisms by inhibiting the CLOCK/BMAL1 complex (Kelleher et al., 2014). CLOCK and BMAL1 heterodimers also stimulate the expression of a large number of clock control genes (CCGs), many of which are associated with the tumor microenvironment (TME) (Xu et al., 2021). Angiogenic factor expression in TME decreases when CLOCK shRNA is knocked down and increases when CLOCK is overexpressed in colorectal cancer cells, according to genetic studies (Wang et al., 2017). CLOCK and PER3 gene expression fluctuates rhythmically in cancer cells in mouse models of BC and is associated with the infiltration of immune inflammatory cells such as macrophages (Wu et al., 2019; Ramos et al., 2020).

The current study suggests that disruption of circadian rhythms is important in the onset and progression of BC (Chi et al., 2017; Angelousi et al., 2019). Circadian rhythm disturbances associated with shift work and exposure to evening light, for example, increase the incidence of BC by 19% and 12%, respectively (Ijaz et al., 2013; He et al., 2015). Furthermore, it has been demonstrated that the circadian rhythm-associated ARNTL2 gene is a distinct prognostic factor in triple-negative breast cancer (Wang et al., 2021). Therefore, it is certain that circadian genes can be used as biomarkers to predict BC prognosis, but current studies on their role in BC are still sparse and one-sided, and the molecular mechanisms of action applicable to broad BC warrant further investigation.

The field of cancer immunotherapy has been forever changed by ICIs that block immunological checkpoints such cytotoxic T lymphocyte-associated antigen 4 (CTLA-4), programmed death 1 (PD-1) and its ligand (PD-L1), and PD-1 ligand 2 (PD-L2). (Bagchi et al., 2021). The mechanism of ICIs for cancer treatment is primarily to reactivate T-lymphocytes and boost the body’s immune system, resulting in anti-tumor activity (Jardim et al., 2021). As a result, identifying biomarker genes associated with immune checkpoints such as CTLA-4 and PD-1 is critical for improving tumor prognosis. Although clock genes have been linked to T-cell depletion (CD8 and CD4 T-cell) and the upregulation of immunosuppressive molecules such as PD-L1 and CTLA-4 (Wu et al., 2019; Zhou J et al., 2020), more research is needed to determine the mechanism of action between circadian genes and immune checkpoints associated with BC prognosis.

Given that, we developed a risk assessment model based on gene expression data and clinical data from The Cancer Genome Atlas (TCGA) to screen circadian rhythm-related genes (CRRGs) of prognostic value in BC patients and validate it in the GEO datasets. Extensive analysis was then used to determine the prognostic value of 18 CRRGs. Finally, the relationship between CRRGs and immune checkpoints was investigated, allowing our CRRGs risk model to guide the prognosis and treatment of BC.

## 2 Materials and methods

### 2.1 Data source and processing

**FIGURE 1:**
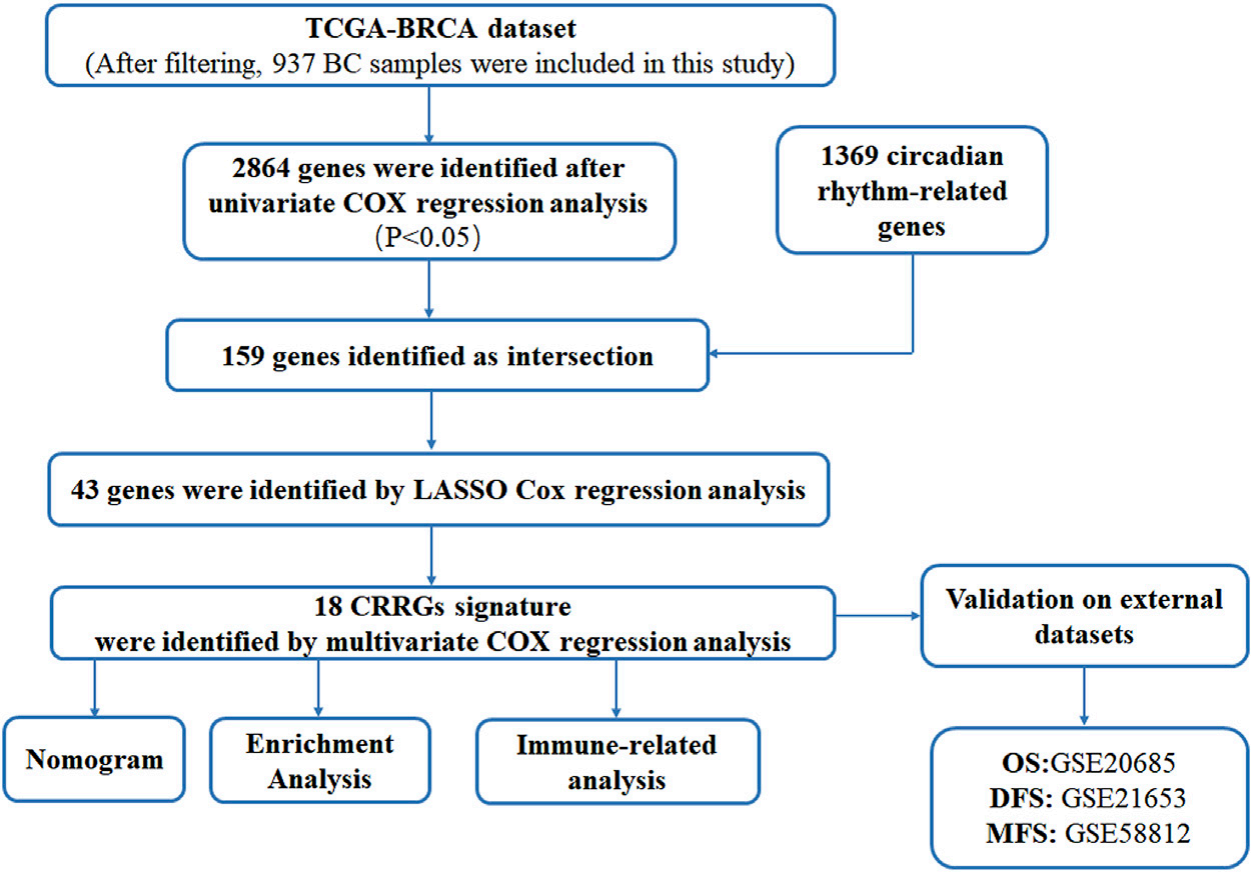
Flow diagram of this study.

We downloaded mRNA sequencing data and clinical information of BC patients from the Cancer Genome Atlas (TCGA) database as a training dataset (TCGA-BRCA; https://portal.gdc.cancer.gov/) Data integration and processing were as follows :1) normal samples were removed; 2) samples with a survival time less than 30 days were screened; 3) only one sample was retained for each patient, and 4) genes expressed in more than half of the samples were retained. After careful screening, 937 BC samples were used for follow-up studies. Gene expression profiles and clinical information of BC patients in the GSE21653, GSE20685, and GSE58812 datasets were used as test data sets from Gene Expression Omnibus (GEO) data (https://www.ncbi.nlm.nih.gov/geo/) downloaded and processed according to the same filtering criteria. Single-cell RNA sequencing (scRNA-seq) data (GSE188600) from triple-negative BC patients were also downloaded from the GEO database for verification. The specific data download and processing process is included in Supplementary Table S1. Moreover, a total of 1369 CRRGs about *Homo sapiens* were obtained from the Circadian Gene Data Base (GCDB, http://cgdb.biocuckoo.org/). These genes have been used as the basis for further research. The detailed flow chart of the research design is shown in Figure 1.

### 2.2 Construction of CRRGs risk score model

In this study, we first performed a univariate COX regression analysis on genes expressed in TCGA-BRCA, then identified genes with *p* < 0.05 as overall survival (OS)-related genes. To find candidate genes, these genes were crossed with 1369 CRRGs. The least absolute contraction and selection operator (LASSO) regression was then used to determine non-zero coefficients, allowing us to eliminate potential predictors and select the best OS-related genes while avoiding model overfitting. Finally, multivariate Cox regression was used to identify and calculate correlation coefficients for candidate genes involved in the final modeling. The associated risk score of the prognostic CRRGs was equal to the product of the prognostic rhythm-related gene’s expression and its coefficient.

### 2.3 Validation of CRRGs risk score model

As previously stated, the training dataset was TCGA-BRCA, while the test dataset was GSE20685 for OS of BC. Based on the median calculated risk scores associated with rhythm genes, samples in each dataset were divided into high-risk and low-risk groups. The Kaplan-Meier survival analyses were then plotted to investigate significant differences in patient survival. Furthermore, receiver operating characteristic (ROC) curves for 1, 2, and 3 years were generated in R software to calculate the area under the ROC curve (AUC) values for each prediction model to further evaluate the model’s efficiency and accuracy. Simultaneously, disease-free survival (DFS) and metastasis-free survival (MFS) of BC were validated using outcome events and time in GSE21653 and GSE58812, respectively, and related graphs were created. In addition, validation of scRNA-seq for BC was performed in the GSE188600 dataset.

### 2.4 Construction of a prognostic normogram

Clinical information was first extracted from the TCGA-BRCA dataset, including age, stage, and risk group. Univariate and multivariate COX regression analyses were then performed to identify independent prognostic factors affecting OS in BC patients. The corresponding operations were then performed in the validation set databases (GSE21653, GSE20685, and GSE58812) to make our extracted information universally convincing (due to missing data, the Stage was only validated in GSE20685). To visualize the Cox regression results, we used the “rms” R package to generate a Normogram that included CRRGs and clinical variables. Age, TNM stage, and CRRGs risk groups were three independent predictors. The scoring criteria were developed based on the magnitude of their regression coefficients, allowing 1-year, 2-year, and 3-year OS probabilities to be calculated. Calibration curves were used to visually demonstrate the consistency of the Normogram model at 1, 2, and 3 years.

### 2.5 Functional enrichment analysis

Differentially expressed genes (DEGs) between high- and low-risk groups were first identified by the “limma” package with screening criteria of |log FC| > 1 and *p*-value_ t < 0.05. This DEG list was then used for gene ontology (GO) analysis and Kyoto Encyclopedia of Genes and Genomes (KEGG) pathway analysis for TCGA-BRCA to explore possible biological functions and signaling pathways. GO analysis included biological process (BP), cellular composition (CC), and molecular function (MF) (*p* < 0.05 was statistically significant). The results of the GO analysis and KEGG pathway were visualized using the R package “ggplot2”.

To further explore the potential molecular mechanisms of rhythm-related genes in breast cancer formation and progression, we performed a gene set enrichment analysis (GSEA) on the BC dataset in the TCGA database to explore the enrichment pathways associated with rhythm genes in high-risk and low-risk groups. “c2. cp.kegg.v7.4. symbols” was chosen to be used for our analysis.

### 2.6 Immuno-infiltration analysis

CIBERSOFT was used to explore the relationship between high- and low-risk groups based on candidate gene characteristics and the abundance of each immune cell type. Information on the 22 immune cell types can be downloaded from the attachment to the previous article (https://www.nature.com/articles/nmeth.3337#MOESM207). Box plots were then plotted using the ggplot2 R package to visually represent differences in abundance. Additionally, we investigated the relationship between CCGRs and immunological checkpoints (PD1, PDL2, and CTLA4) as well as the connection between risk scores for CCGRs and immune checkpoints.

### 2.7 Predicting the response of BC patients to ICIs

To investigate the response of BC patients in high- and low-risk groups to ICIs treatment, we obtained immunophenotype score (IPS) data for BC from The Cancer Immunome Atlas (TCIA, https://tcia.at/), which quantifies tumor immunogenicity scores ranging from 0 to 10. IPS values are associated with tumor immunogenicity and can be used to predict patient response to ICIs treatment (Charoentong et al., 2017). Furthermore, we compared the expression of immune checkpoints in high- and low-risk groups.

### 2.8 Statistical analysis

Statistical analyses were carried out using R (version 4.2.0) and SPSS 26.0. The independent prognostic value of clinical characteristics and risk groups associated with CRRGs was described using univariate and multifactorial COX. Pearson correlation coefficients were used to examine the relationship between CRRGs and immune checkpoints. The presence of two-sided *p* values less than 0.05 was considered statistically significant.

## 3 Results

### 3.1 Identification of prognostic characteristics of CRRGs

**FIGURE 2:**
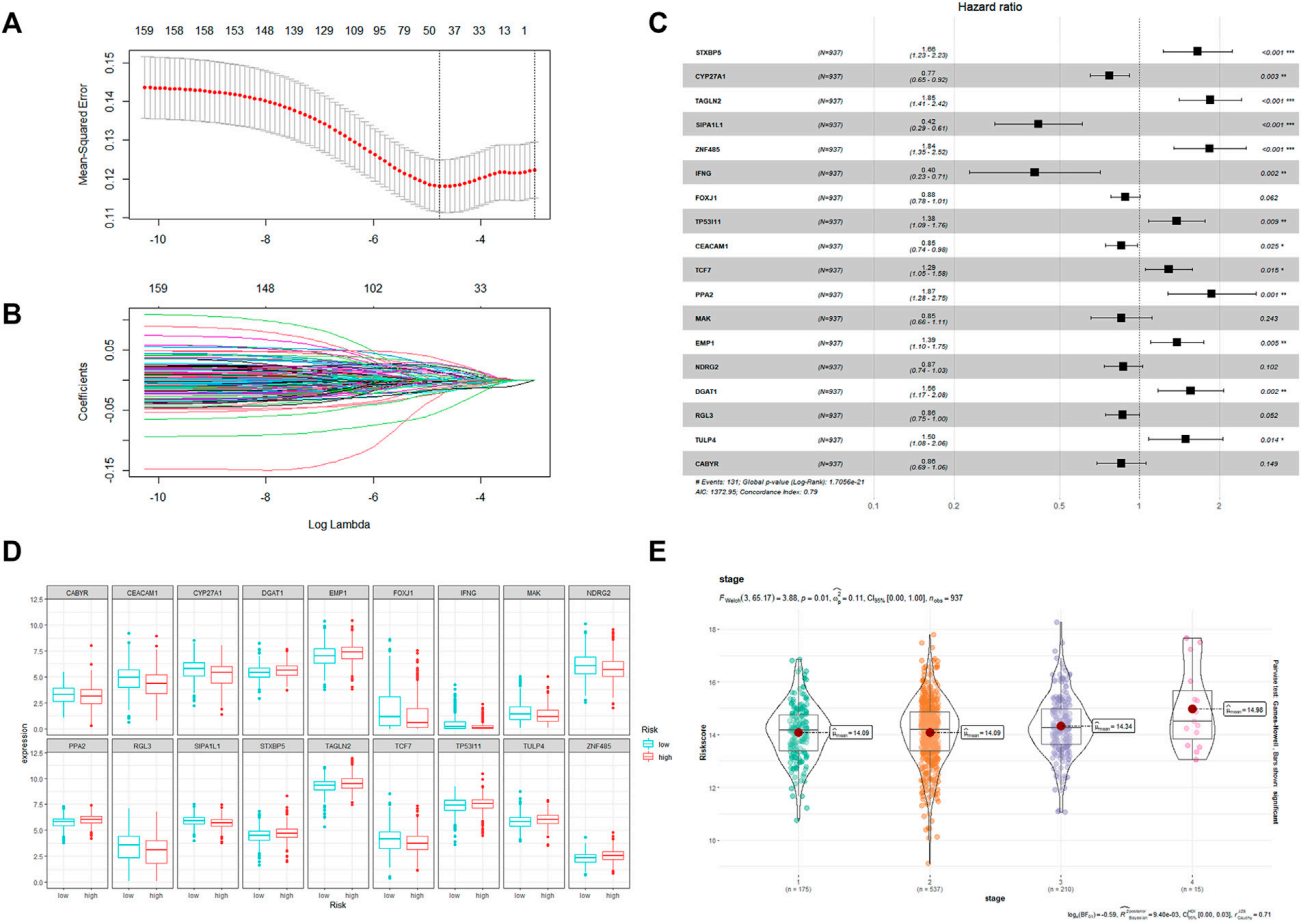
**(A)** The LASSO Cox regression model was utilized to identify CRRGs. **(B)** Select range of the optimal parameter (lambda) in the LASSO Cox regression model. **(C)** The coefficient of the selected CRRGs. **(D)** Expression of 18 CRRGs in the high and low risk groups. **(E)** The distribution of the CRRGs risk score in different TNM stages of TCGA-BRCA dataset.

The flow chart in Figure 1 shows that we finally constructed a prognostic model associated with 18 CRRGs, which include STXBP5, CYP27A1, TAGLN2, SIPA1L1, ZNF485, IFNG, FOXJ1, TP53I11, CEACAM1, TCF7, PPA2, MAK, EMP1, NDRG2, DGAT1, RGL3, TULP4, and CABYR. Figures 2A, B shows the LASSO regression process. The relationship between these 18 candidate genes and the core clock genes is shown in Supplementary Figure S3. A prognostic risk score formula was developed based on the linear combination of CRRGs expression levels and the weighted regression coefficients of multiple Cox regression analysis: Risk score = 0.557*STXBP5-0.189*CYP27A1+0.70*TAGLN2-0.814*SIPA1L1+0.747*ZNF485–1.192*IFNG-0.139*FOXJ1+0.314*TP53I11 0.238*CEACAM1+0.373*TCF7+0.577*PPA2-0.253*MAK+0.333*EMP1-0.220*NDRG2+0.366*DGAT1-0.152*RGL3+0.350*TULP4-0.237*CABYR. These candidate genes were categorized into risk types (STXBP5, TAGLN2, ZNF485, TP53I11, TCF7, PPA2, EMP1, DGAT1, TULP4) and protective types (CYP27A1, SIPA1L1, IFNG, CEACAM1, RGL3), with HR > 1 (*p* < 0.05) being associated with poor prognosis and HR < 1 (*p* < 0.05) being associated with better prognosis (Figure 2C). Interestingly, NDRG2 and CABYR were present and expressed at higher levels in the normal group despite FOXJ1, MAK, and EMP1 not being in the human protein atlas (HPA; https://www.proteinatlas.org/), proving that these two genes are also protective. In addition, the expression differences of the other 15 CRRGs in normal and BC tissues were indexed in the HAP database and shown in Supplementary Figure S1. Clinical stage was found to be positively correlated with risk score (Figure 2E), with a statistically significant difference (*p* = 0.01). Based on the median risk score, patients were divided into high-risk and low-risk groups, with the expression of 18 candidate genes in TCGA in both groups shown in Figure 2D and the expression in the three validation sets shown in Supplementary Figure S6.

### 3.2 Validation of model prediction effects of CRRGs

**FIGURE 3:**
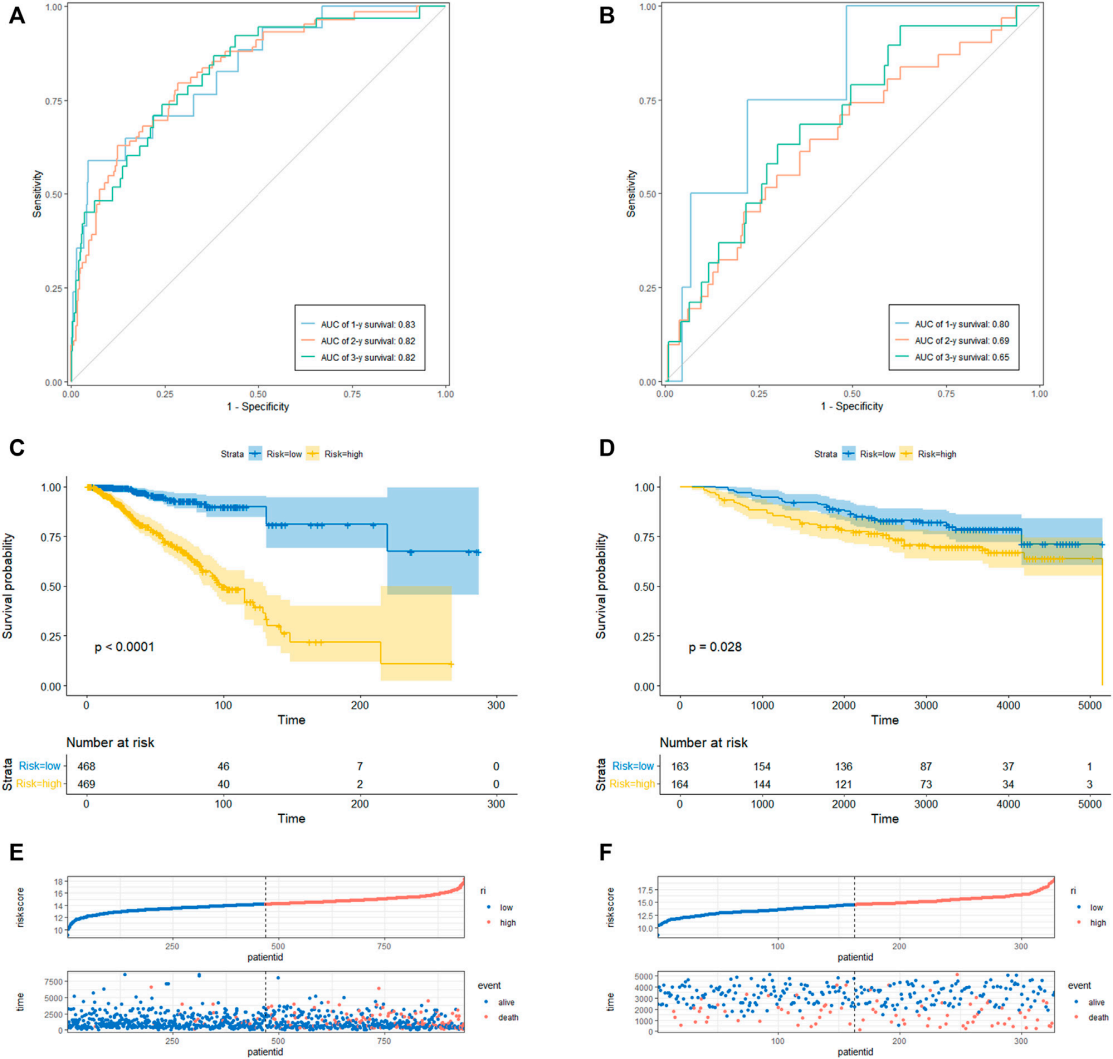
CRRGs signature associated with BC patient’s overall survival (OS). **(A)** The predictive value for the 1-y, 2-y and 3-y OS in TCGA-BRCA dataset. **(B)** The predictive value for the 1-y, 2-y and 3-y OS in GSE20685 dataset. **(C)** The OS between the CRRGs high- and low-risk groups in TCGA-BRCA dataset. **(D)** The OS between the CRRGs high- and low-risk groups in GSE20685 dataset. **(E)** The risk plot of the CRRGs signature in TCGA-BRCA dataset. **(F)** The risk plot of the CRRGs signature in GSE20685 dataset.

**FIGURE 4:**
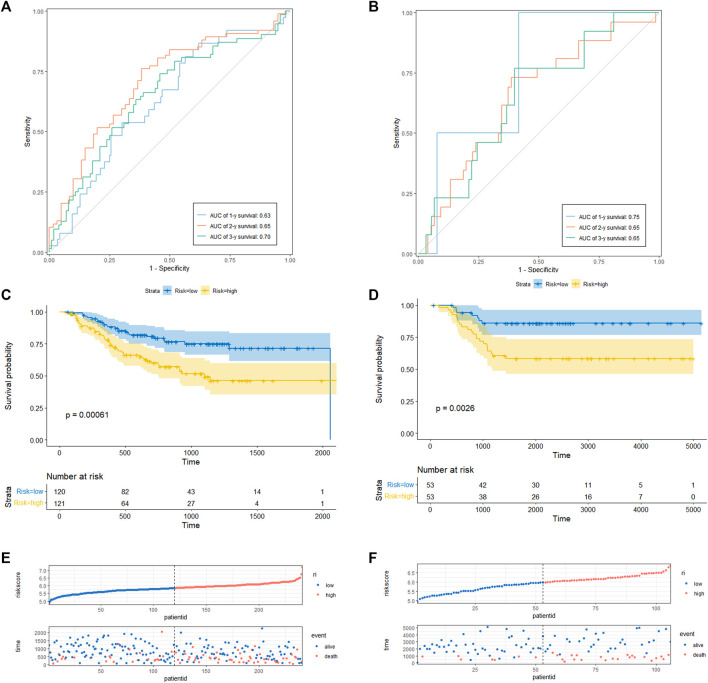
CRRGs signature associated with BC patient’s disease-free survival (DFS) and metastasis-free survival (MFS). **(A)** The predictive value for the 1-y, 2-y and 3-y DFS in GSE21653 dataset. **(B)** The predictive value for the 1-y, 2-y and 3-y MFS in GSE58812 dataset. **(C)** The DFS between the CRRGs high- and low-risk groups in GSE21653 dataset. **(D)** The MFS between the CRRGs high- and low-risk groups in GSE58812 dataset. **(E)** The risk plot of the CRRGs signature in GSE21653 dataset. **(F)** The risk plot of the CRRGs signature in GSE58812 dataset.

To determine the prediction accuracy of the 18 candidate genes, OS validation was performed using GSE20685 datasets, and DFS and MFS validation were performed using two other external datasets (GSE21653 and GSE58812), respectively. The AUCs for 1-year, 2-year, and 3-year survival in the TCGA test set were 0.83, 0.82, and 0.82 (Figure3A), whereas in GSE20685, GSE21653, and GSE58812, the AUCs for 1-year, 2-year, and 3-year survival were 0.80, 0.69, and 0.65; 0.63, 0.65, and 0.70; 0.75, 0.65, and 0.65, respectively (Figure 3B, Figures 4A, B).

Kaplan-Meier survival analysis showed that patients in the high-risk group had a significantly shorter survival time than those in the low-risk group (TCGA: *p* < 0.0001; GSE20685: *p* = 0.028; GSE21653: *p* = 0.00061; GSE58812: *p* = 0.0026) (Figure 3C, D; Figures 4C, D). Moreover, risk scores were negatively correlated with survival time in all four data sets (Figures 3E, F, Figures 4E, F). In addition, the results of scRNA-seq are shown in Supplementary Figure S4, 5.

### 3.3 Construction of a nomogram for CRRGs and clinical characteristics

**FIGURE 5:**
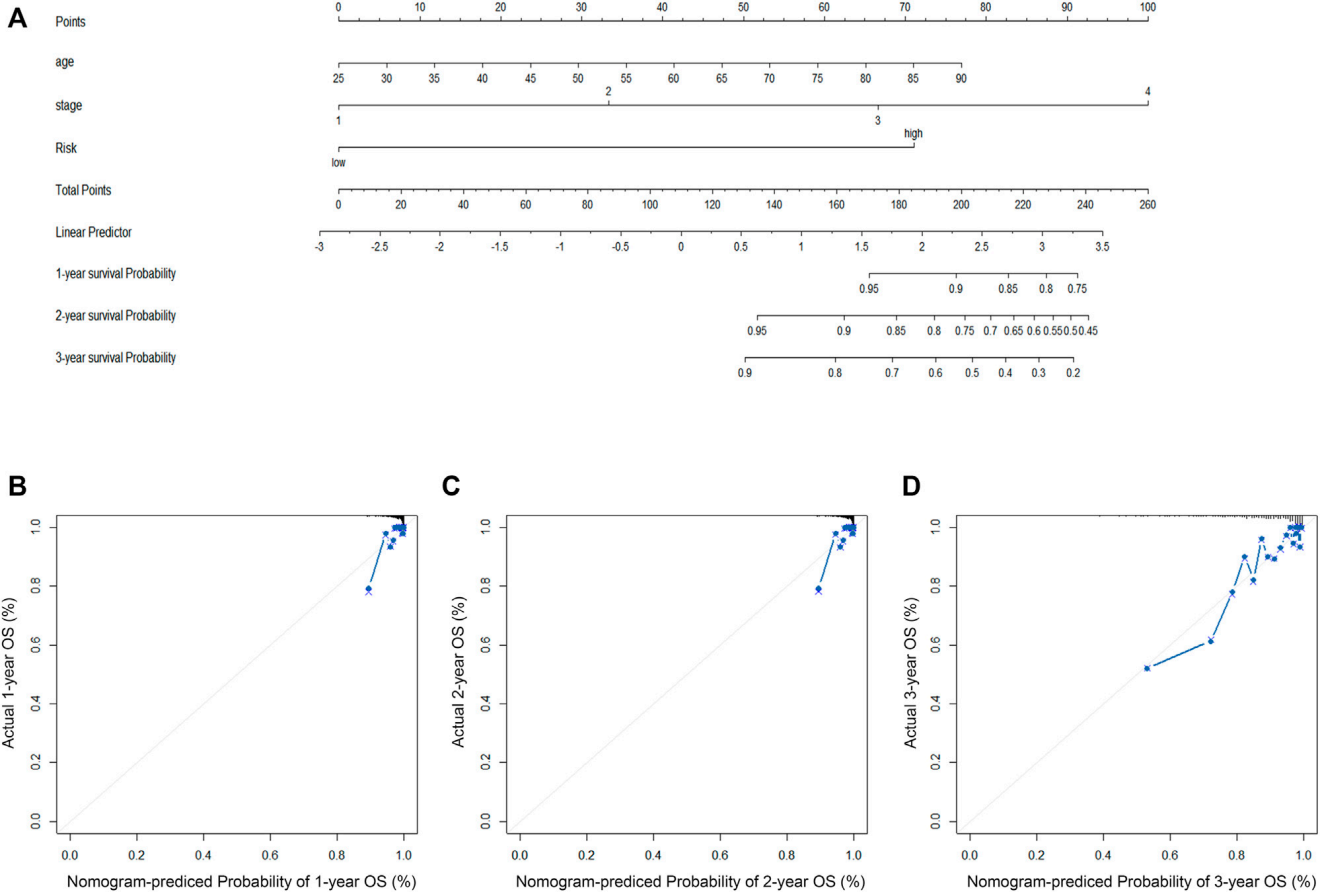
**(A)** The construction of OS predictive nomogram for TCGA-BRCA patients. **(B–D)** 1-year, 2-year, and 3-year calibration curves of the nomogram combined model in TCGA-BRCA dataset.

First, the risk score and other clinical parameters were used as covariates in univariate and multivariate Cox proportional risk regression models. The risk group was found to perform well as a prognostic factor affecting the survival cycle of BC patients in both the TCGA and validation set databases. Table 1; Table 2 also showed the results for age and stage. As a result, we selected the three variables listed above to create follow-up nomograms. The findings revealed that as the total score increased, the survival time decreased (Figure 5A). The calibration curve revealed that the nomogram model had high predictive accuracy for OS at 1, 2, and 3 years and significantly overlapped with the 45-degree angle line (Figures 5B–D). Additionally, Supplementary Table S1 displayed the survival times (OS, MFS, and DFS) of the three external validation sets (GSE21653, GSE20685, and GSE58812) in relation to CRRGs.

**TABLE1:** Univariate and multivariate Cox regression analysis of clinical characteristics and survival of BC patients in TCGA Cohort.

	Univariate analysis		Multivariate analysis	
	HR (95% CI)	*p*-value	HR (95% CI)	*p*-value
Age	1.036 (1.022–1.051)	0.000	1.028 (1.014–1.042)	0.000
Stage (I/II)	1.502 (0.866–2.606)	0.148	1.872 (1.072–3.271)	0.028
Stage (I/III)	2.871 (1.610–5.120)	0.000	3.708 (2.056–6.687)	0.000
Stage (I/IV)	15.385 (7.370–32.115)	0.000	18.892 (8.978–39.754)	0.000
Risk (High/Low)	0.155 (0.096–0.251)	0.000	0.155 (0.096–0.252)	0.000

Abbreviations: HR: hazard ratio; CI: confidence interval.

**TABLE 2:** Univariate and multivariate Cox regression analysis of clinical characteristics and survival of BC patients in Validation set Cohort.

	Univariate analysis		Multivariate analysis	
	HR (95% CI)	*p*-value	HR (95% CI)	*p*-value
GSE20685 (OS)				
Age	1.006 (0.982–1.031)	0.620	0.999 (0.975–1.023)	0.919
Stage (I/II)	3.141 (1.300–7.590)	0.011	2.835 (1.152–6.978)	0.023
Stage (I/III)	1.077 (0.401–2.895)	0.883	1.191 (0.440–3.223)	0.730
Stage (I/IV)	1.816 (0.795–4.150)	0.157	1.697 (0.735–3.916)	0.216
Risk (High/Low)	0.503 (0.299–0.847)	0.010	0.582 (0.339–0.999)	0.049
GSE21653 (DFS)				
Age	1.001 (0.983–1.019)	0.927	0.997 (0.979–1.015)	0.997
Risk (High/Low)	0.444 (0.276–0.716)	0.001	0.439 (0.271–0.710)	0.001
GSE58812 (MFS)				
Age	1.061 (1.025–1.098)	0.001	1.054 (1.020–1.089)	0.002
Risk (High/Low)	0.293 (0.125–0.685)	0.005	0.324 (0.138–0.764)	0.010

Abbreviations: HR: hazard ratio; CI: confidence interval; OS: overall survival; DFS: disease-free survival; MFS: metastasis-free survival.

### 3.4 Enrichment analysis based on CRRGs risk model

**FIGURE 6:**
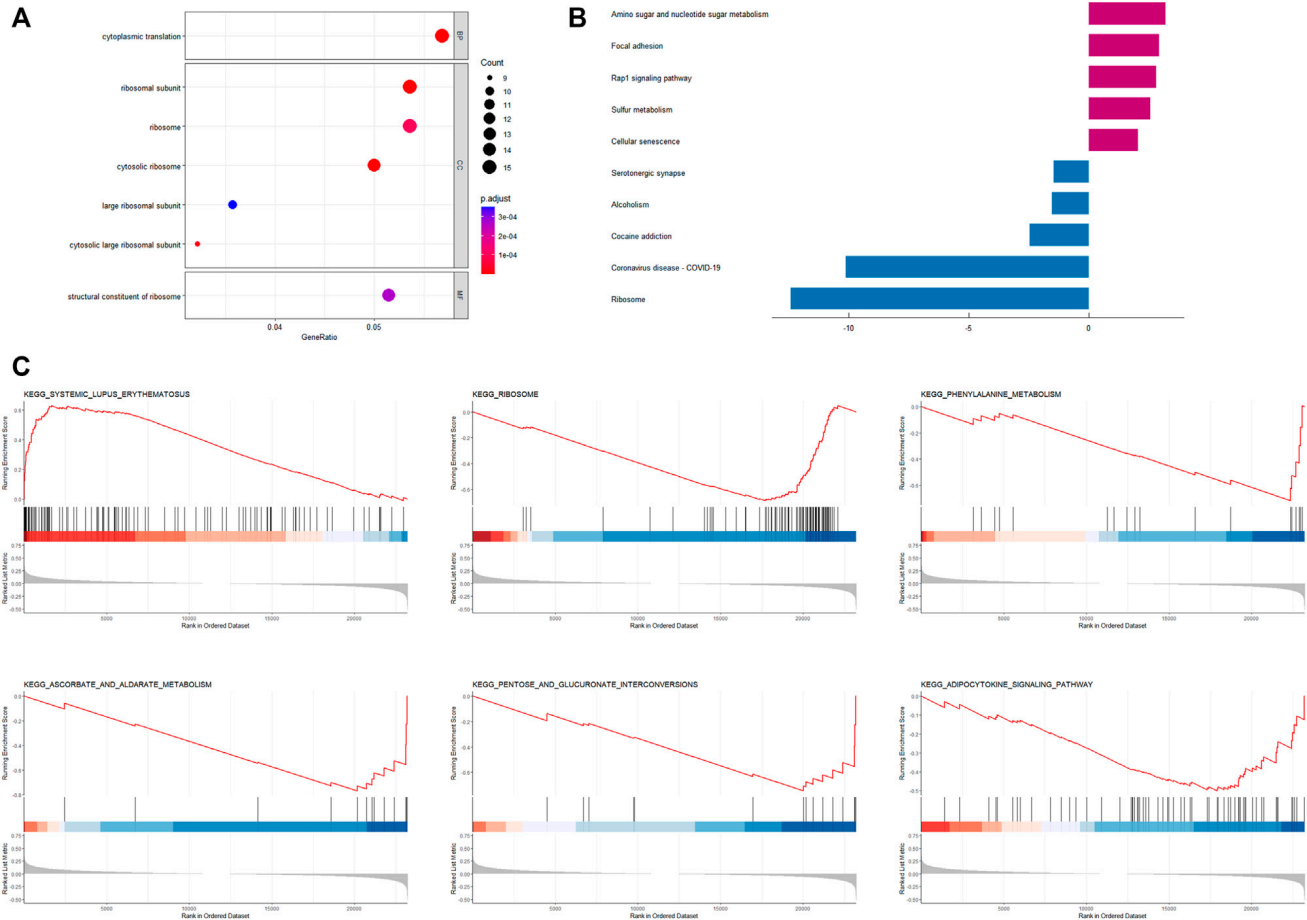
Enrichment analysis based on CRRGs risk model **(A)** Bubble plot for GO enrichment analysis based on CRRGs. **(B)** Bar chart of KEGG enrichment analysis based on CRRGs. **(C)** Gene enrichment analysis of CRRGs in the TCGA-BRCA dataset.

Based on the results of the “limma” variance analysis, we identified 354 differentially expressed genes (DEGs), including 193 up-regulated DEGs and 161 down-regulated DEGs. The bubble diagram shows the terms of these DEGs in GO-BP, GO-CC and GO-MF (Figure 6A). GO functional annotation shows that these DEGs are mainly involved in cytoplasmic translation, ribosomes, and ribosome structural composition. A two-way bar graph showing the top five pathways of KEGG upregulation and downregulation (Figure 6B). GSEA showed that the significantly enriched pathway in the high-risk group was systemic lupus erythematosus, whereas in the low-risk group it was the adipocytokine signaling pathway, ascorbate and aldehyde metabolism, pentose and glucuronide interconversion, phenylalanine metabolism, and ribosomes (Figures 6C–H). Furthermore, using a risk-score model, we investigated the genetic alterations of these CRRGs and discovered that they are relatively conserved evolutionarily (mutation rates of 1% or less) (Supplementary Figure S2), which is consistent with the current study (Cederroth et al., 2019).

### 3.5 Immuno-infiltration analysis in CRRGs risk groups

**FIGURE 7:**
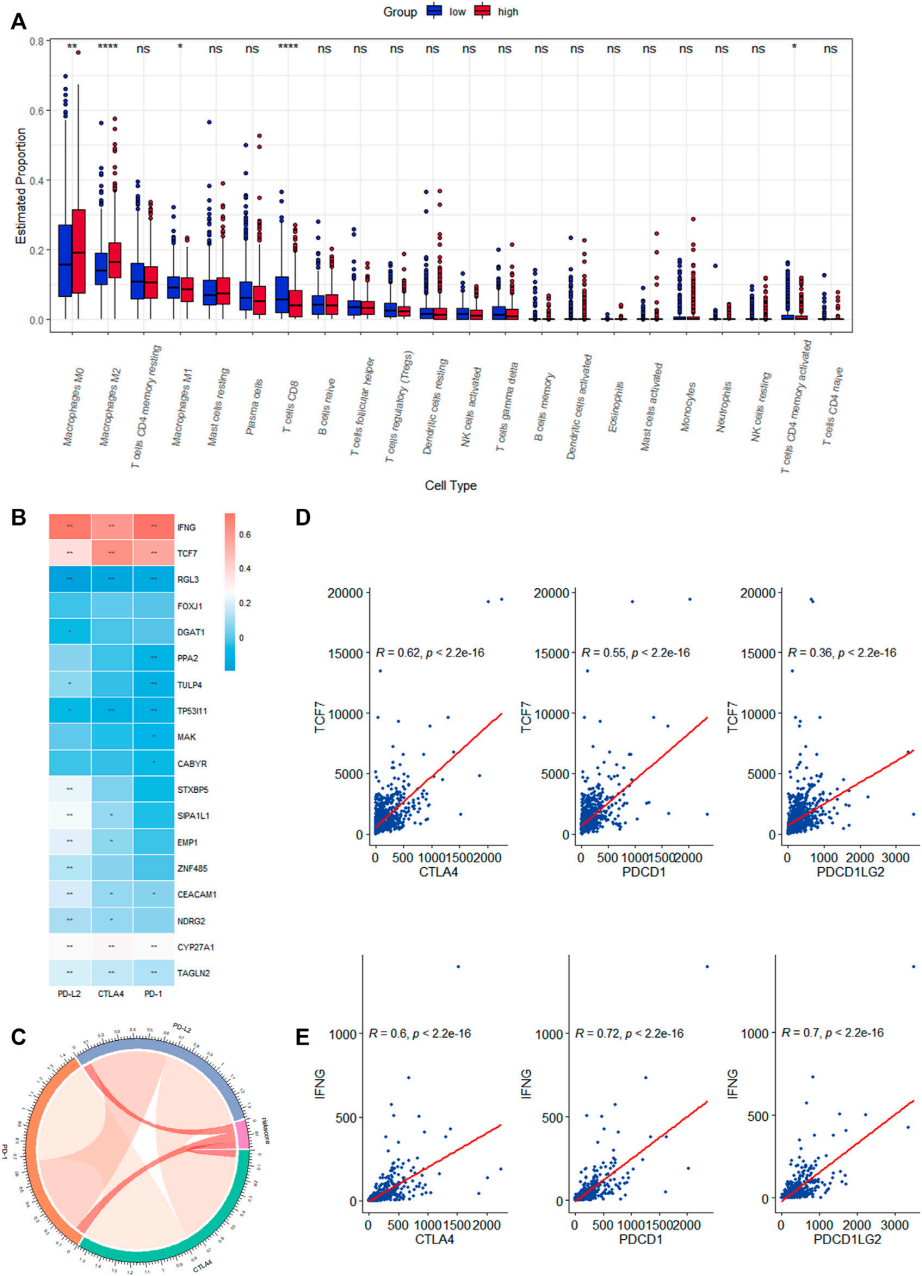
Correlation analysis of the expression of eighteen CRRGs in BC with the level of complex immune infiltration. **(A)** Differences of the abundance of 22 immune cells between high- and low-risk groups in the TCGA-BRCA dataset. **(B)** Correlation heat map between immune checkpoints and eighteen CRRGs. **(C)** Chord plot of the correlation between immune checkpoints and risk scores. **(D)** Correlation between TCF7 expression levels in BC and three immune checkpoints. **(E)** Correlation between IFNG expression levels in BC and three immune checkpoints.

We investigated the level of infiltration of 22 immune cells in various risk groups, and box plots revealed that the level of immune infiltration of T-cell CD8 and T-cell CD4 memory activated was significantly lower in the high-risk group, while immunosuppressive cells such as macrophages M0 and M2 were significantly higher (Figure 7A). The high-risk group appeared to have an immunosuppressive tumor microenvironment filled with a large number of immunosuppressive cells, which was consistent with a poor prognosis. The correlation heat map between CRRGs and immune checkpoints is shown in Figure 7B, which shows that IFNG and TCF7 are strongly correlated with immune checkpoints (Figures 7D, E). These two genes were also shown in scRNA analysis to be distributed mainly in T-cell (Supplementary Figure S4E). The chord plot of the correlation between risk scores and immune checkpoints is represented in Figure 7C.

### 3.6 Response of patients in the CRRGs risk groups to ICIs treatment

**FIGURE 8:**
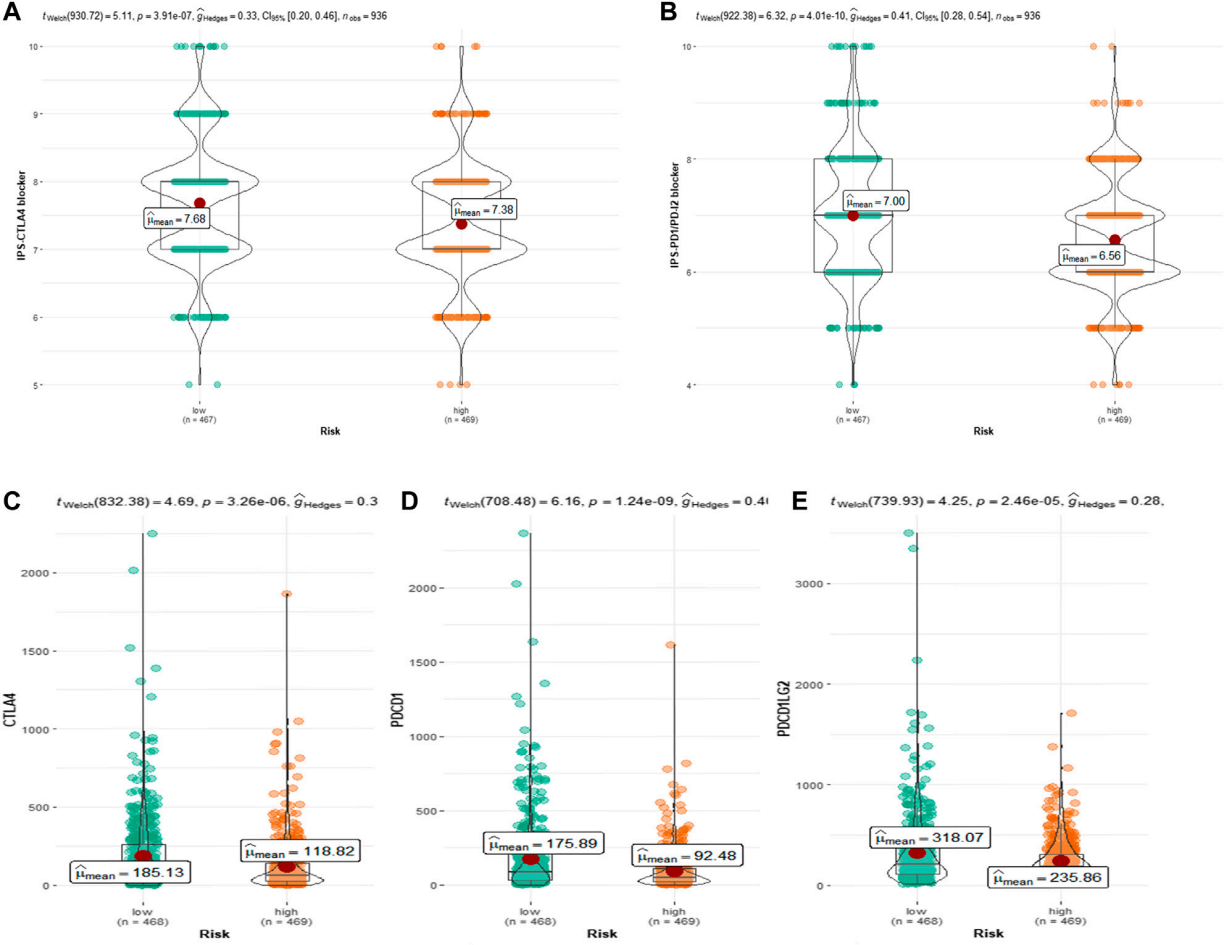
**(A)** Relative probability of response to CTLA-4 treatment in low-risk and high-risk groups. **(B)** Relative probability of response to PD-1/PD-L2 treatment in low-risk and high-risk groups. Expression of **(C)** CTLA-4, **(D)** PD-L1 and **(E)** pdcd1LG2(PD-L2) in low- and high-risk groups.

Because the TCGA-BRCA dataset lacked information on ICIs treatment, we used two IPS-valued subtypes (IPS- PD-L1/PD-1/PD-L2 blocker and IPS-CTLA-4 blocker) as proxies for response to anti-PD-1/PD-L1 and anti-CTLA-4 treatment in BC patients. In the CRRGs prediction model, the low-risk group had a higher relative probability of receiving anti-PD-1/PD-L1 and anti-CTLA-4 treatment (Figures 8A, B). The findings imply that patients with low CRRG risk scores may be candidates for ICIs treatment. Furthermore, we compared immune checkpoint expression levels between the high-risk and low-risk groups. Patients with low risk had significantly higher levels of PD-1, PD-L2, and CTLA-4 (Figure 8C).

## 4 Discussion

Circadian rhythm is shaped at the molecular level by oscillations of clock genes, which maintain body homeostasis by controlling cell proliferation, cell metabolism, and various gene expression pathways (Shi et al., 2020). Disrupting the regular cell cycle, circadian rhythm disorders are accompanied by variable expression of CRRGs and are linked to the start of several chronic illnesses, including cancer and metabolic disorders (Masri and Sassone-Corsi, 2018; Sulli et al., 2018). Circadian rhythm disorders are classified as possible human carcinogens by the International Agency for Research on Cancer (IARC) in 2007 (Group 2A) (Straif et al., 2007). A growing body of evidence suggests that disruption of circadian rhythms is closely linked to the development of BC (Hansen, 2017; Salamanca-Fernández et al., 2018). However, the significance of CRRGs in the prognosis of BC patients, as well as the underlying molecular mechanisms, is rarely recognized and studied in clinical research and practice, and even less so when translated to the bedside. As a result, our research is attempting to take a significant step in this direction.

Using the TCGA-BRCA dataset, we developed a prognostic risk score model for CRRGs. We validated the model’s prognostic predictive efficacy with three GEO datasets, and Kaplan-Meier analysis and time-ROC curves confirmed that the model could accurately predict OS, RFS, and MFS in BC patients. This model contains 18 CRRGs, some of which have been linked to BC development, invasion, and prognosis. Particularly, not only is CYP27A1 abundant in macrophages (Hansson et al., 2003), but it also catalyzes the conversion of cholesterol to 27-hydroxycholesterol (27HC), which promotes the growth of estrogen receptor-dependent tumor cells (Nelson et al., 2013). TAGLN2 is a major cause of paclitaxel resistance in BC patients, and new strategies to reverse paclitaxel resistance in BC patients include various drugs and pathways to reduce TAGLN2 expression (Cai et al., 2014; Wang et al., 2019). TP53I11 inhibits the epithelial-mesenchymal transition (EMT) of BC cells, preventing cancer metastasis (Xiao et al., 2019; Zhai et al., 2022). In our study, CEACAM1 and NDRG2 were protective factors for BC prognosis (HR < 1), which is consistent with the previous consensus that they are tumor suppressors (Yang et al., 2015). Not only were their levels reduced in BC cells, but also the expression of NDRG2 was found to be negatively correlated with PD1 expression, suggesting that increasing NDRG2 expression could activate T-cell proliferation to counteract tumor invasion (Lee et al., 2021). FOXJ1 and EMP1 have also been shown to predict BC prognostic regression and outcome (Sun et al., 2014; Zhou X et al., 2020). Furthermore, other CRRGs, such as PPA2, DGAT1, and CABYR, have been shown to be involved in the oncogenic effects of other cancers and to affect patient prognosis, despite the fact that related studies are uncommon in BC (Luo et al., 2007; Cheng et al., 2020; Zhu et al., 2021).

Immune cells play an important role in the tumor microenvironment (TME). The disruption of the circadian rhythm results in the loss or reversal of the daily pattern and cytokine levels of M0 and M2 macrophages, which exhibit immunosuppression and homeostasis and are beneficial to tumor cell proliferation (Chanmee et al., 2014; Aiello et al., 2020). For example, the high-risk group was found to be positively correlated with immunosuppressive cells such as M2 macrophages in two newly published studies on the prediction of triple-negative BC prognosis by cell death patterns (Pu et al., 2022; Zou et al., 2022). In our prognostic model, M2 macrophage levels were significantly higher in the high-risk group than in the low-risk group, while T-cell CD8 and T-cell CD4 memory activation levels were significantly lower. Because the Kaplan-Meier survival analysis revealed that the high-CRRGs risk group had shorter survival times, it suggests that immune infiltration is an important factor influencing the prognosis of BC patients. Therefore, in order to confirm the critical function of CRRGs in BC immunity, we further investigated immunological checkpoints. Cancer cells are well known for activating immune checkpoints with immunosuppressive functions in order to suppress the body’s immune function and thus achieve rapid proliferation (Darvin et al., 2018). ICI is a monoclonal antibody that binds to and inhibits CTLA4 or PD1, two key signal pathways involved in T-cell activation and failure (Wright et al., 2021). Although ICIs have been shown to improve survival time in advanced patients with non-small cell lung cancer and melanoma (Duma et al., 2019; Lozano et al., 2022), their efficacy in BC patients is low (Keenan and Tolaney, 2020). This method raises the issue of identifying patients who respond to treatment. According to our findings, higher immune checkpoint expression in the low-risk group is consistent with lower IPS in the low-risk group, and together they represent a better response to ICIs in low-risk patients. Furthermore, our findings show that IFNG and TCF7 in CRRGs are linked to three immune checkpoints, which is consistent with previous research (Bassez et al., 2021; Xie et al., 2022). As a result, we can predict the response of BC patients to ICIs using our CRRGs model, which is extremely important for promoting the treatment and development of ICIs in BC patients.

Ribosomal pathways were found to be enriched in GO, KEGG, and GSEA in our study. This result is easy to understand given the known molecular function and mechanism of ribosomes. Ribosome biogenesis is a common sign of cell growth and proliferation because ribosomes are required for protein production (Turi et al., 2019). Upregulation of ribosome biogenesis during G1/S arrest can facilitate tumor metastasis-related EMT (Prakash et al., 2019). In a recent study, CRISPR activation was used to screen the whole genome of circulating tumor cells (CTC) from BC patients for genes that promote tumor metastasis. They discovered that genes encoding ribosomal proteins were overrepresented (Ebright et al., 2020). As a result, high ribosome levels can be used as a marker of BC invasion and metastasis, which are linked to a poor prognosis of BC. In addition, several other cancer-related pathways, such as the adipocytokine signaling pathway and cellular senescence, were enriched in the low-risk group. Adiponectin, which is secreted by fat cells in the breast microenvironment, inhibits the growth for cancer cells (Chung et al., 2017). Cellular senescence is a permanent state of cell cycle arrest that is thought to be a tumor-inhibiting mechanism (Calcinotto et al., 2019). These enrichment pathways are consistent with the conclusion that patients in the low-risk group have a longer survival time.

Although our study had some positive results, its limitations should not be overlooked. Since this is a retrospective study, it is inevitable that there would be missing data and selection bias. Second, this CRRGs model is based on a publicly accessible database. Although it performed well in three GEO datasets, its predictive ability needs to be validated further through randomized controlled experiments. Finally, we used IPS values to simulate patient reactions to ICIs. While there is evidence for a correlation, there are some differences between IPS and patient responses to ICIs treatment.

## 5 Conclusion

In conclusion, the risk assessment model based on 18 CRRGs can effectively evaluate BC patients’ prognosis and immunotherapy effect. Patients with a low-risk score have a better prognosis and response to ICIs treatment. These CRRGs, we believe, should be prospectively validated as promising prognostic biomarkers for BC and used to guide immunotherapy strategies in the future.

## Data Availability

The original contributions presented in the study are included in the article/Supplementary Material, further inquiries can be directed to the corresponding authors.
