# Supplementary material for: A circadian rhythm-related gene signature for prognosis, invasion and immune microenvironment of breast cancer
Source: Front Genet. 2023 Jan 5;13:1104338. doi: 10.3389/fgene.2022.1104338 (PMC9849377; doi:10.3389/fgene.2022.1104338)
Supplement: Supplementary file 2 [file DataSheet1.docx]

Supplementary Material

A Circadian rhythm-related gene signature for prognosis invasion and immune microenvironment of breast cancer

**Mei-Huan (given name) Wang (family name)^1^, Xiao (given name) Liu (family name)^1^, Qian (given name) Wang (family name)^1, 2*^, Hua-Wei (given name) Zhang (family name)^1, 2*^**

*** Correspondence:**Hua-Wei Zhang (slyyzhw@163.com)

Qian Wang (wangqian122411@126.com)

This file includes:

Figure S1-S6

## Supplementary Figures


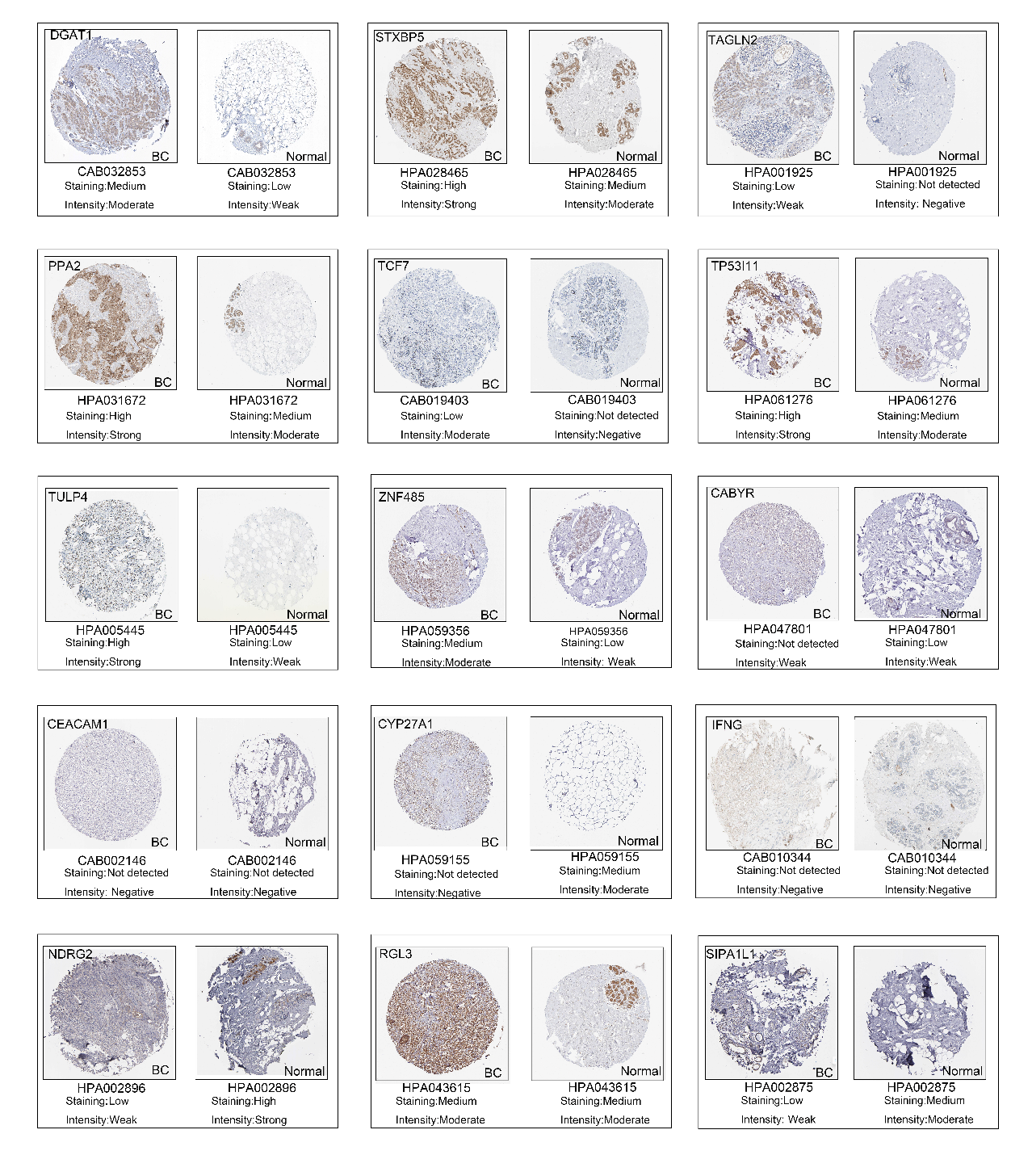


**Supplementary Figure 1.** The representative protein expressions of the prognostic CRRGs in breast normal and cancer tissue.

**
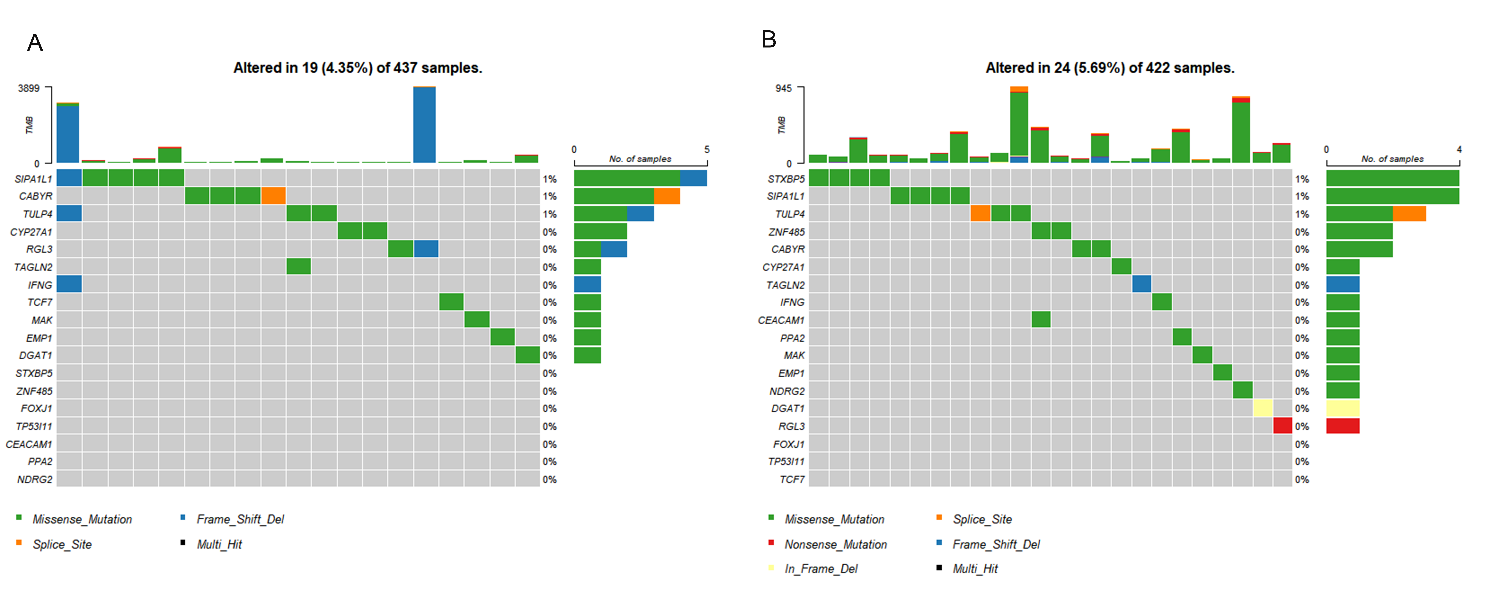
**

**Supplementary Figure 2.** Profiles of mutations in high (A) - and low (B) -risk groups of BC patients.


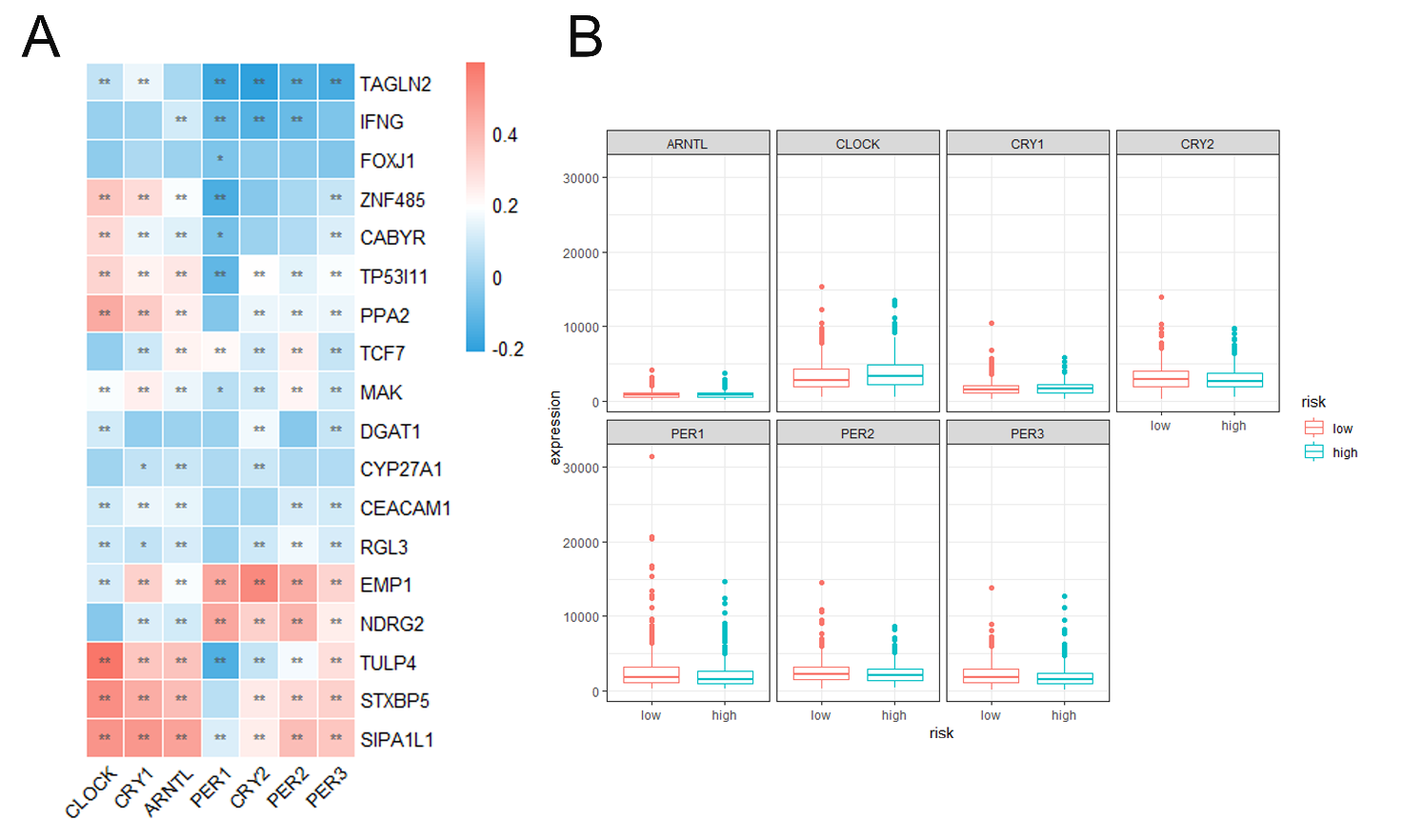


**Supplementary Figure 3.** (A) Heat map of 18 CRRGs in relation to core clock genes. (B) Boxplot of differences in expression of core clock genes between different risk groups constructed based on 18 CRRGs.


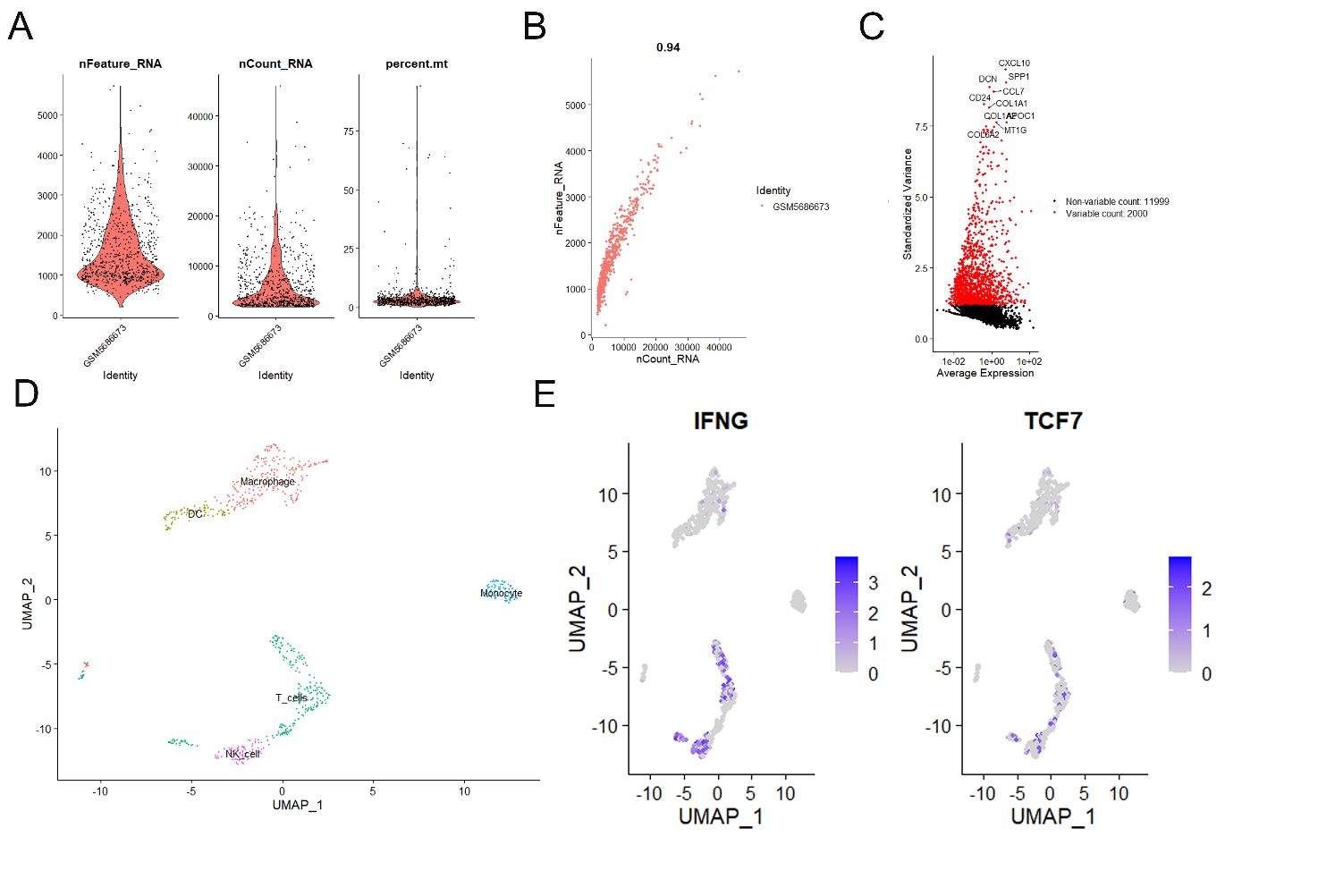


**Supplementary Figure 4.** Identify and annotate 8 cell clusters according to marker genes. (A) After quality control of the scRNA-seq data, 729 cells from BC from GSM5686673 samples were included in the analysis. (B) Examination of the correlation analysis between sequencing depth and detected genes, Pearson correlation coefficient of 0.94. (C) The variance of 13,999 genes was analyzed, and the results showed that 2,000 genes marked with red dots had high variability and 11,999 genes marked with black dots had low variability. (D) Based on marker genes, cell clusters annotated by singleR. (E) Gene expression analysis of IFNG and TCF7.


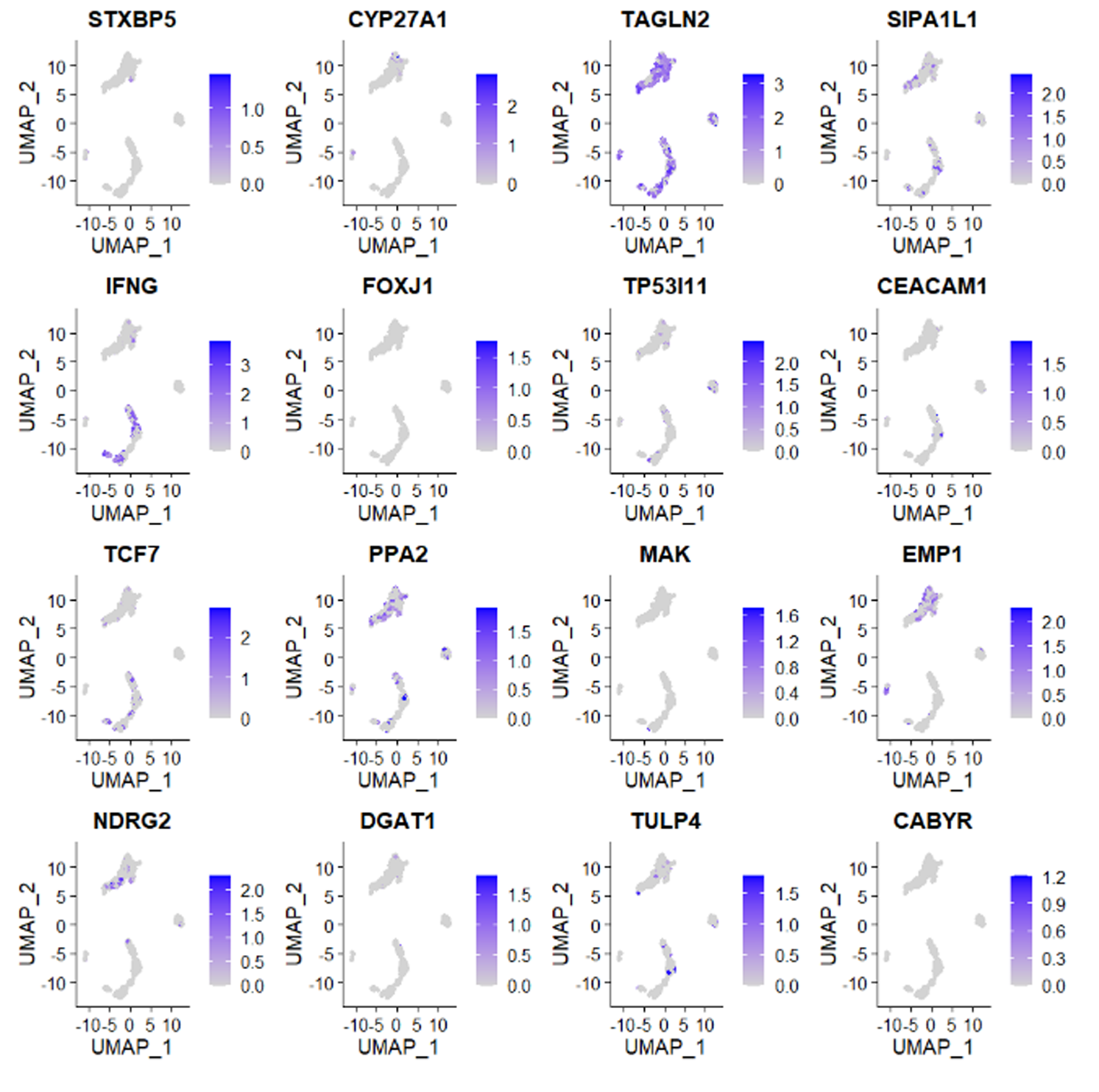


**Supplementary Figure 5.** Expression analysis of 18 CRRGs


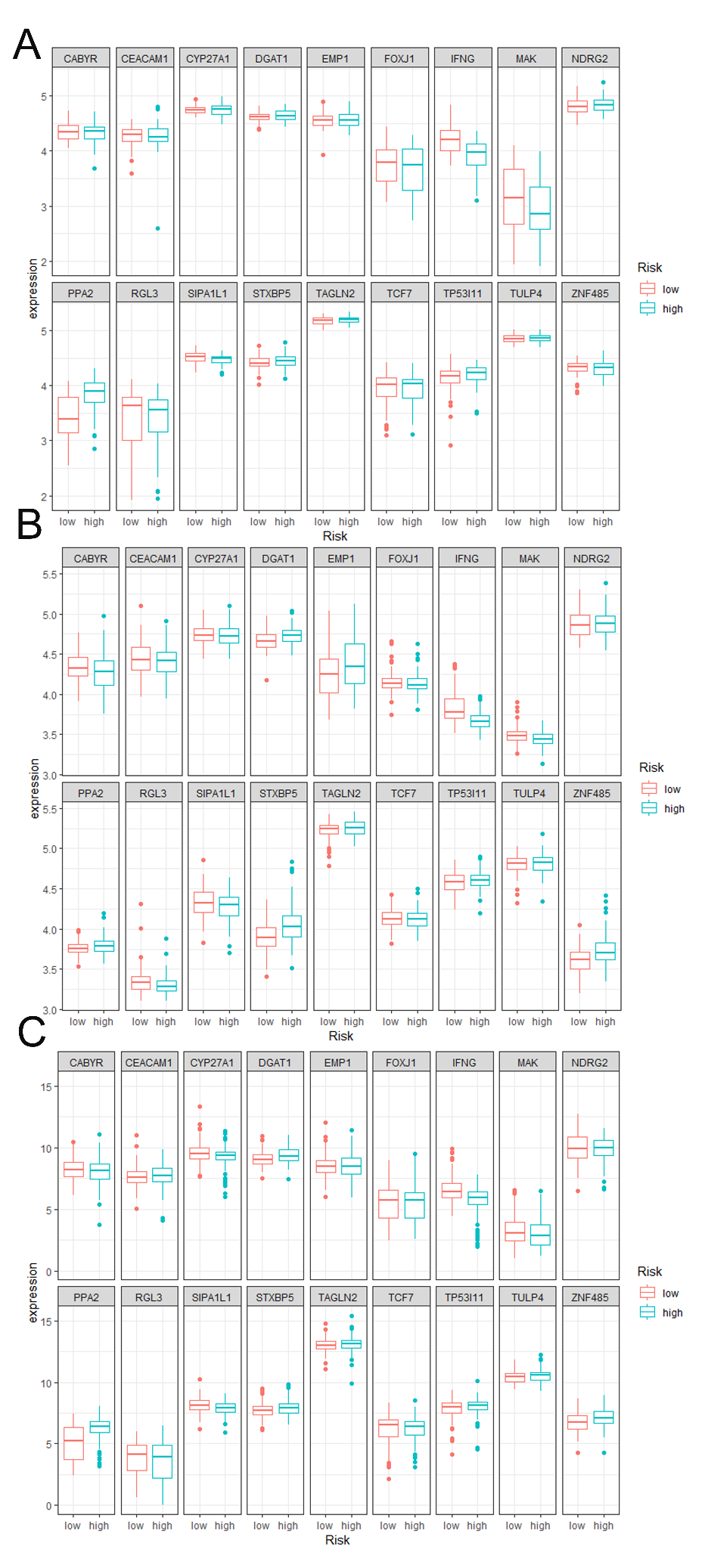


**Supplementary Figure 6.** Boxplots of 18 CRRGs expressed in different risk groups. (A) GSE58812. (B) GSE21653. (C) GSE20685.

| **Supplementary** Table1: Univariate and multivariate analyses of patient survival with CRRGs in validation set databases | | | | |
| --- | --- | --- | --- | --- |
| **Variable** | **Univariate analysis** | | **Multivariate analysis** | |
|  | **HR (95% CI)** | ***P*‐Value** | **HR (95% CI)** | ***P*‐Value** |
| **GSE20685 (OS)** |  |  |  |  |
| CYP27A1 | 0.646 (0.490-0.851) | 0.002 | 0.707 (0.525-0.952) | 0.022 |
| IFNG | 0.659 (0.540-0.805) | 0.000 | 0.712 (0.564-0.899) | 0.004 |
| TP53I11 | 1.872 (1.213-2.890) | 0.005 | 1.720 (1.092-2.710) | 0.019 |
| TCF7 | 0,735 (0.592-0.913) | 0.005 | 0.867(0.680-1.106) | 0.250 |
| **GSE21653 (DFS)** |  |  |  |  |
| TAGLN2 | 26.834 (2.359-305.184) | 0.008 | 55.033 (4.495-673.720) | 0.002 |
| IFNG | 0.088 (0.017-0.443) | 0.003 | 0.063 (0.012-0.330) | 0.001 |
| NDRG2 | 0.030 (0.201-0.047) | 0.030 | 0.168 (0.037-0.762) | 0.021 |
| **GSE58812 (MFS)** |  |  |  |  |
| IFNG | 0.178 (0.067-0.472) | 0.001 | NA | NA |
| Abbreviations: HR: hazard ratio; CI: confidence interval. NA: not available; OS: overall survival; DFS: disease-free survival; MFS: metastasis-free survival. | | | | |
